# Resveratrol enhances HBV replication through activating Sirt1-PGC-1α-PPARα pathway

**DOI:** 10.1038/srep24744

**Published:** 2016-04-21

**Authors:** Yixian Shi, Yongjun Li, Chenjie Huang, Lixiong Ying, Jihua Xue, Haicong Wu, Zhi Chen, Zhenggang Yang

**Affiliations:** 1 State Key Lab of Diagnostic and Treatment of Infectious Diseases, Collaborative Innovation Center for Diagnosis and Treatment of Infectious Disease, the First Affiliated Hospital, Zhejiang University School of Medicine, Hangzhou 310003, China; 2 Department of Pathology, the First Affiliated Hospital, College of Medicine, Zhejiang University, Hangzhou 310003, China; 3 Department of Infectious Disease, the First Affiliated Hospital of Anhui Medical University, Hefei 230022, Anhui, China; 4 Department of Hepatobiliary Medicine, Fuzhou General Hospital of Nanjing Military Command, Fuzhou 350025, Fujian, China

## Abstract

The population of hepatitis B combined with a number of metabolic disorders is increasing significantly. Resveratrol (RSV) has been used as a preclinical drug for the treatment of the metabolic disorders. However, the impact of RSV on HBV replication remains unknown. In this study, the HBV-expressing hepatocelluar carcinoma cell line and mouse model created by hydrodynamic injection of viral DNA were used. We found that RSV activates Sirt1, which in turn deacetylates PGC-1α and subsequently increases the transcriptional activity of PPARα, leading to the enhanced HBV transcription and replication *in vitro* and *in vivo*. In addition, we found that this pathway is also required for fasting-induced HBV transcription. Taken together, this study identifies that RSV enhances HBV transcription and replication especially acting on the core promoter, which depends on Sirt1-PGC-1α-PPARα pathway. We conclude that RSV may exacerbate the progression of hepatitis B and that patients with hepatitis B infection should be cautious taking RSV as a dietary supplement.

Hepatitis B virus (HBV) is a small enveloped DNA virus, which has chronically infected more than 350 million people worldwide and caused about 1 million people death per year due to HBV-associated liver pathologies^1^. Current first-line drugs for HBV treatment include interferon α (IFNα) and nucleoside or nucleotide analogues. While these drugs can minimize HBV replication, they are unable to cure hepatitis B. Therefore, protecting HBV patients from recurring of high HBV DNA levels to minimize the liver damages is still one of the most important strategies for managing this infectious disease.

Increasing evidence shows that, as a result of sedentary behaviors and poor dietary choices, more and more people suffer metabolic syndromes^2^. It correlates with an increased risk of diabetes, cardiovascular diseases and stroke^3^,^4^,^5^,^6^. HBV patients have also been subjected to the metabolic disorders and, at present, the prevalence of combined metabolic syndrome and chronic HBV infection in the general population is around 0.99–1.74%^7^,^8^,^9^. Although the interaction between chronic hepatitis B and metabolic diseases is controversial, the presence of both chronic HBV infection and metabolic syndrome further increases the risk of cirrhosis and hepatocellular carcinoma (HCC)^10^,^11^,^12^,^13^.

Resveratrol (trans-3,5,40-trihydroxystilbene, RSV) is a polyphenol found in a variety of plant species. RSV has been shown to exert beneficial effects across species and various disease models. It prevents or slows down the progression of a variety of illnesses, especially metabolic diseases^14^,^15^,^16^. Recently it has been shown that RSV has impacts on various viral infections via different pathways. For instance, RSV has been shown to antagonize the respiratory syncytial virus infection in the lung via inhibiting the TRIF/TBK1/IRF-3 complex^17^. RSV was also found to be effective in treating influenza virus^18^ and severe acute respiratory syndrome (SARS)^19^. RSV has also been shown to synergize with several antiretroviral drugs in the treatment of human immunodeficiency virus (HIV)^20^ and herpes simplex viruses (HSV)^21^. In an *in vivo* study, 25% RSV cream can achieve the same efficacy in the treatment of HSV-1-induced skin lesions on the abraded epidermis of SKH1 mice as 5% acyclovir cream^22^. In addition, RSV has been confirmed to enhance replication of hepatitis C virus, which indicates that RSV was not suitable as an antioxidant therapy for chronic hepatitis C^23^.

However, the effect of RSV on hepatitis B virus remains unclear. In this study, we investigated the role of RSV in regulation of HBV replication. Surprisingly, we found that RSV administration dramatically up-regulated HBV DNA production in HBV-expressing cells and the mouse model via activating the Sirt1-PGC-1α-PPARα pathway.

## Results

### RSV increases HBV replication *in vitro* and *in vivo*

**Figure 1:**
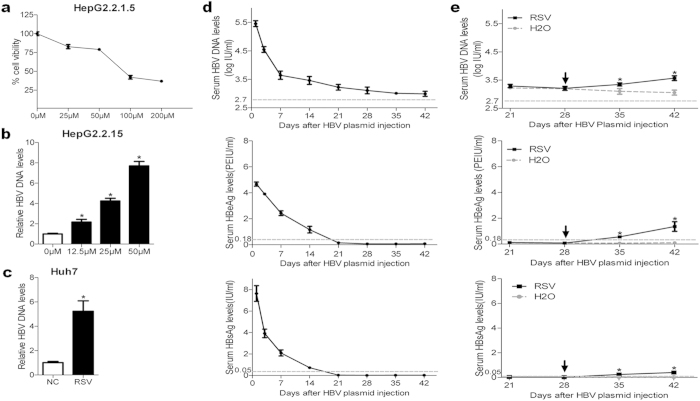
RSV increases HBV replication in HBV-expressing liver cells and mouse model. (**a**) Cell viability in HepG2.2.15 cells treated with RSV. **(b)** RSV increased supernatant HBV DNA contents in HepG2.2.15 cells in a dose-dependent manner. **(c)** RSV increased supernatant HBV DNA contents in Huh7 cells transfected with pHBV. Expression data was normalized to vehicle control (NC). **(d)** Establishment of the mouse model with HBV expression. The sera of mice injected with 10 μg pHBV were collected at the time points indicated and quantified for HBV DNA (top), HBeAg (middle) and HBsAg (bottom). **(e)** Daily RSV treatment began at day 28 after hydrodynamic injection and lasted for 2 weeks. Serum HBV DNA (top), HBeAg (middle) and HBsAg (bottom) were shown at indicated times. *p < 0.05 with n = 6/group.

We first tested the toxicity of RSV in HepG2.2.15 cells and determined the optimal dose for the later treatment. The 50% inhibitory concentration (IC50) of cell viability for RSV treatment was 106.3 μM and then the 50 μM was an effective concentration chosen for the following experiments of RSV (Fig. 1a). To explore the impact of RSV on HBV replication in HepG2.2.15 cells, equivalent numbers of cells were exposed to 50 μM RSV or control vehicle for 72 hours and HBV DNA in the culture medium was measured by real-time qPCR. We found that RSV treatment dose-dependently upregulated HBV DNA levels by nearly 8 times higher than the control (Fig. 1b). Same results were obtained in HepG2 (data not show) and Huh7 cells transiently transfected with 1.3mer HBV genomic DNA (Fig. 1c). We then established an HBV replication mouse model with hydrodynamic injection of 1.3mer HBV genomic DNA (10 μg/per mouse) according to a previous study^24^. After injection, the serum HBV DNA number in mice remained at a low level of [10^3^] IU/ml for more than one month (Fig. 1d), which is consistent with the previous data^25^. After treatment of RSV (100 mg/kg/d) via daily oral gavage of RSV for 2 weeks, the serum HBV DNA levels were significantly increased and maintained at a higher level towards the end point of the drug administration (Fig. 1e), suggesting that RSV is a strong stimulator for HBV DNA replication.

### RSV activates the HBV core promoter

**Figure 2:**
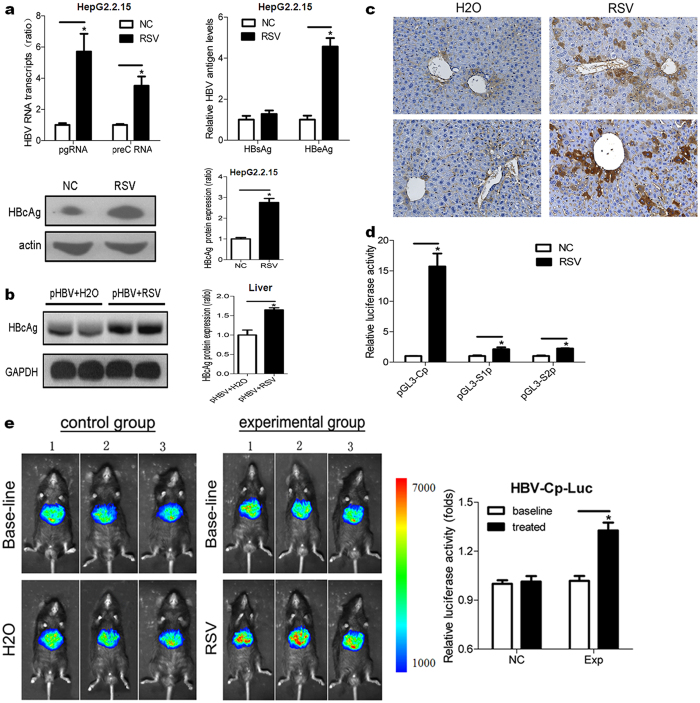
RSV enhances HBV replication through promoting the viral core promoter in HBV-expressing liver cells and mouse model. (**a)** RSV enhanced HBV RNA (pgRNA, preC RNA) and protein (supernatant HBsAg, HBeAg and HBcAg) expression in HepG2.2.15 cells. The cropped blots are used in the figure and full-length blots are presented in Supplementary Figure 1a. **(b,c)** The expression levels of HBcAg in the mouse liver were shown by using Western blot **(b)** and immunohistochemistry ((**c)**, HBcAg Brown staining. magnification 20×). The cropped blots are used in the figure and full-length blots are presented in Supplementary Figure 1b. **(d)** Effect of RSV treatment on HBV promoters. The luciferase activity was assessed after 72-hour RSV treatment posttransfection. **(e)** In vivo luciferase analysis of mice was observed after administration of water (control group, NC) or RSV (experimental group, Exp). Representative images for visualization of luciferase activity (left) and their quantitative analysis data (right) were shown. The gels have been run under the same experimental conditions. *p < 0.05 with n = 6 /group.

To further investigate the mechanism by which RSV upregulates HBV replication, we measured the pregenomic RNA (pgRNA) and preC RNA in HepG2.2.15 cells by real-time qPCR, HBcAg expression by Western blot, and the HBsAg and HBeAg contents released into the culture medium by ELISA. We found that RSV treatment increased not only pgRNA and preC RNA levels by almost 6-fold and 4-fold respectively, but also HBeAg and HBcAg levels by nearly 5-fold and 3-fold respectively, with a slight increase in HBsAg expression (Fig. 2a), suggesting that the transcription of HBV DNA was activated by the RSV treatment, especially at the region of viral core promoter. Our *in vivo* experiments further supported these observations. RSV treatment (100 mg/kg/d) for 2 weeks increased HBsAg and HBeAg levels in circulation (Fig. 1e) as well as HBcAg expression in the liver (Fig. 2b,c), indicating that the viral core promoter plays a key role in the process. We then measured the activities of the preS1, preS2 and core promoters respectively in HepG2 cells. We found that while RSV treatment increased the core promoter activity by nearly 16-fold, it activated preS1 and preS2 promoters with much less potency (Fig. 2d). To further confirm the results *in vivo*, 6 μg of 1.3× HBV-Cp-Luc plasmid DNA was hydromatically injected into the mice. 72 hours later, the mice were treated with RSV for 20 minutes. The activity of the core promoter in mice liver was measured by the animal imaging system in real time. We found that RSV increased the luciferase signals in the liver by nearly 1.3-fold, compared with the vehicle control (Fig. 2e). These results suggest that RSV promotes HBV replication by activating the HBV core promoter.

### RSV enhances HBV transcription via PPARα/RXRα activation

**Figure 3:**
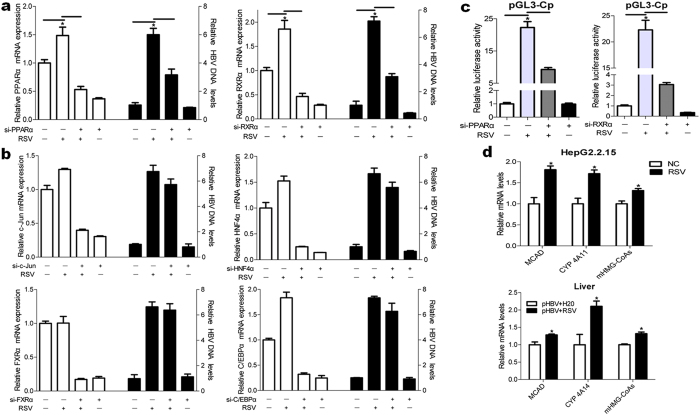
RSV-induced HBV transcription is dependent on PPARα/RXRα activation. **(a,b)** Gene expressions of various transcription factors (empty bars) and the corresponding supernatant HBV DNA levels (filled bars) in HepG2.2.15 cells were measured after RSV treatment following RNAi of indicated genes. **(c)** Effects of PPARα or RXRα silencing on viral core promoter in HepG2 cells treated with RSV. **(d)** RSV induced the PPARα/RXRα targets. The mRNA expression of the PPARα/RXRα targets in HepG2.2.15 cells (top) and mouse liver (bottom) was measured by RT-qPCR. All experiments were repeated at least three times with consistent results. Bar graphs represent the means ± SEM, n = 3 (*p < 0.05).

Efficient transcription of HBV genes requires a number of ubiquitous transcription factors^26^. To identify the transcription factor(s) involved in the activation of the core promoter by RSV, we knocked down various HBV-related transcriptional factors including Jun Proto-Oncogene *(c-Jun)*, peroxisome proliferator-activated receptor, alpha*/*retinoid x receptor, alpha *(PPARα/RXRα),* CCAAT/Enhancer binding protein (C/EBP), alpha *(C/EBPα),* farnesoid x -activated receptor *(FXRα), and* hepatocyte nuclear factor 4, alpha *(HNF4α)* in HepG2.215 cells by small interfering RNAs (siRNAs). Real-time qPCR data demonstrated that knockdown of PPARα or RXRα abolished RSV-induced HBV replication (Fig. 3a), while knockdown of the others didn’t affect supernatant HBV DNA levels (Fig. 3b). To examine whether PPARα/RXRα could regulate the HBV core promoter, we knocked down PPARα or RXRα by siRNA in HepG2 cells following pGL3-Cp transfection and RSV treatment. Evidently, knockdown of either PPAR or RXR reduced the luciferase signal of the core promoter (Fig. 3c). To further evaluate the activity of PPARα/RXRα affected by RSV treatment, we measured the expression of PPARα/RXRα target genes by RT-qPCR in HepG2.2.15 cells and the mouse liver. RSV stimulated the expression of PPARα target genes promoting fatty acid oxidation (FAO), such as key mitochondrial medium chain acyl-CoA dehydrogenase (*MCAD*), microsome cytochrome *P450* in FAO and mitochondrial 3-hydroxy-3-methylglutaryl-CoA synthase (*mHMG-CoAs*) in ketogenesis (Fig. 3d). Taken together, the activation of PPARα/RXRα on core promoter plays a key role in RSV-induced HBV transcription.

### Sirt1/PGC-1α is required for RSV-induced HBV replication

**Figure 4:**
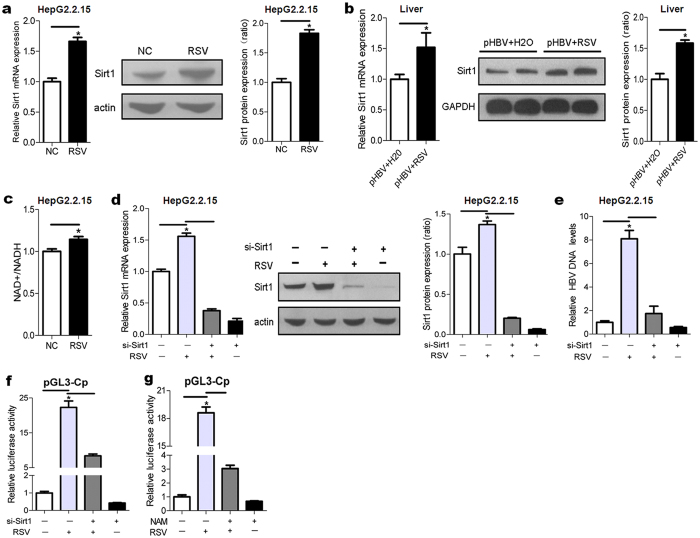
RSV activates Sirt1 and increases HBV replication. (**a,b**) RSV increased Sirt1 mRNA and protein levels in HepG2.2.15 cells after 72-hour treatment **(a)** and the liver of HBV mouse model after 14-day treatment **(b)**. The cropped blots are used in the figure and full-length blots are presented in Supplementary Figure 2a,b. **(c)** Cellular NAD/NADH ratio was examined after RSV treatment for 24 h. **(d)** Knockdown of endogenous Sirt1 in HepG2.2.15 cells. The cropped blots are used in the figure and full-length blots are presented in Supplementary Figure 2c. **(e)** Knockdown of Sirt1 blocked RSV-induced HBV replication in HepG2.2.15 cells. **(f)** Gene silencing of Sirt1 inhibited the effect of RSV on viral core promoter activity in HepG2 cells. **(g)** Sirt1 antagonist NAM markedly blocked RSV-stimulated activity of the viral core promoter. HepG2 cells were cotransfected with pGL3-Cp and pRL-TK plasmids for 24 hours and cells were then treated with RSV (50 μM) or RSV (50 μM) plus NAM (10 mM) for 72 h. The gels have been run under the same experimental conditions. All experiments were repeated at least three times with consistent results. Bar graphs represent the means ± SEM, n = 3 (*p < 0.05).

RSV can act as a positive regulator of sirtuin 1 (Sirt1) to improve the metabolic process of aging, hepatic steatosis and diabetes^27^,^28^,^29^,^30^,^31^. To examine whether Sirt1 is also involved in RSV-induced HBV replication, we measured the expression of endogenous Sirt1 as well as its activity in HepG2.2.15 cells and mouse liver. We found that RSV not only increased Sirt1 mRNA and protein levels (Fig. 4a,b), but also enhanced the ratio of NAD/NADH (Fig. 4c), consistent with the previous reports^32^,^33^. To examine whether Sirt1 contributes to RSV-induced HBV replication, we measured HBV DNA in the culture media from Sirt1 knockdown cells (Fig. 4d). Knockdown of Sirt1 significantly decreased the HBV DNA levels by almost 80% compared to the control (Fig. 4e). The activation of HBV core promoter by RSV was also substantially blocked by Sirt1 knockdown (Fig. 4f). In addition, nicotinamide (NAM), a Sirt1 activity inhibitor, also dramatically reduced RSV-stimulated HBV core promoter activity (Fig. 4g). These data demonstrated an essential role of Sirt1 in RSV-stimulated HBV replication.

**Figure 5:**
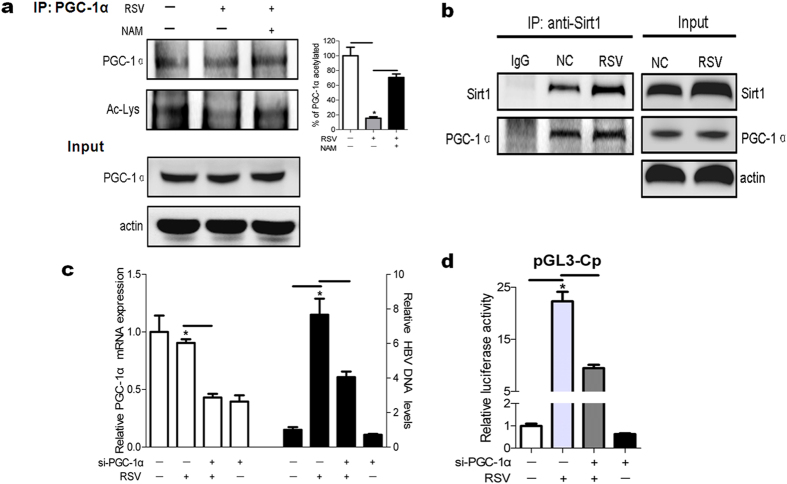
Sirt1 activates PPARα/RXRα via deacetylating PGC-1α. (**a**) Total PGC-1α was immunoprecipitated and its acetylation (Ac-Lys) was examined by western blot analysis. The cropped blots are used in the figure and full-length blots are presented in Supplementary Figure 3a. **(b)** Sirt1 physically interacted with PGC-1a in HepG2.2.15 cells. Cells were extracted and immunoprecipitated with anti-Sirt1 antibody, and the immunocomplexes were analyzed by western blot with anti-PGC-1α antibody. IgG antibody was used as a negative control. The cropped blots are used in the figure and full-length blots are presented in Supplementary Figure 3b. **(c)** Gene silencing of PGC-1α (empty bars) inhibited the effect of RSV on supernatant HBV DNA level (filled bars) in HepG2.2.15 cells. **(d)** PGC-1α knockdown substantially blocked RSV-stimulated activity of the viral core promoter. The gels have been run under the same experimental conditions. Data expressed as mean ± SEM of three independent experiments, n = 3 (*p < 0.05).

Previous studies have demonstrated that in hepatic lipid metabolism, Sirt1 can activate PPARα via deacetylating peroxisome proliferator-activated receptor gamma coactivator 1, alpha (PGC-1α)^34^. To analyze whether PGC-1α is a signal that links Sirt1 to PPARα/RXRα in RSV-induced HBV replication, we examined the PGC-1α acetylation in HepG2.2.15 cells. RSV treatment significantly decreased the acetylation level of PGC-1α protein in HepG2.2.15 cells (Fig. 5a) and increased the interaction between endogenous Sirt1 and PGC-1α (Fig. 5b). Pretreatment of nicotinamide (NAM), a Sirt1 activity inhibitor, restored the acetylated PGC-1α level to that of the untreated group (Fig. 5a). To determine whether PGC-1α is required for RSV-induced HBV replication, we knocked down PGC-1α by siRNA in HepG2.2.15 cells. PGC-1α deficiency caused a significant decrease of HBV DNA in the culture medium compared to the control (Fig. 5c). Similarly, knockdown of PGC-1α significantly reduced RSV-stimulated core promoter activity in HepG2 cells (Fig. 5d). In sum, these findings indicate that Sirt1 activated by RSV directly regulates PGC-1α acetylation, resulting in RSV-induced HBV replication.

### Activation of PPARα is required for fasting-induced HBV replication

**Figure 6:**
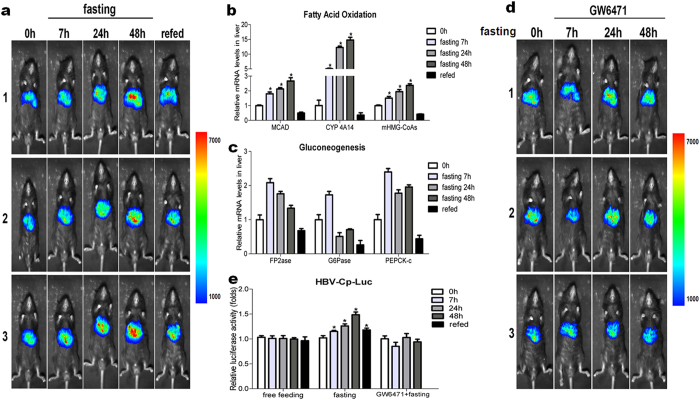
PPARα activation is required for fasting-induced HBV transcription *in vivo*. (**a**) Fasting enhanced the activity of the viral core promoter in mice. *In vivo* luciferase analysis of all mice was performed at baseline and at indicated times of fasting. Shown were the luciferase images of three representative animals from the experimental group at the indicated times of fasting. (**b,c**) Fasting induced the expression of PPARα and HNF4α target genes. The hepatic expression of PPARα target genes (**b**) encoding *MCAD, CYP 4A14 and mHMG-CoAs* and HNF4α target genes (**c**) encoding *FP2ase, G6Pase, PEPCK-c* were measured by RT-qPCR at indicated times of fasting. (**d**) Inhibition of PPARα by GW6471 prevented fasting-induced activity of the viral core promoter. Representative images from the experimental group for visualization of luciferase activity were shown at the indicated times. (**e**) A relative quantitative analysis of the luciferase activity described in (**a**,**d**). *p < 0.05 with n = 6/group.

Calorie restriction has a similar effect on energy metabolism to that of RSV treatment^35^,^36^. It was reported that fasting can induce HBV gene expression through PGC-1α^37^. Given that the fact that PPARα’s transcriptional activity can be activated by fasting^38^,^39^ and the essential role of PGC-1α/PPARα axis in mediating RSV-induced HBV replication, we reckoned that PPARα may be required for fasting-triggered HBV replication. To test this idea, the mice were starved for different time periods after injection of 1.3 × HBV-Cp-Luc plasmid DNA. The core promoter activity, assessed by the luciferase intensity, was time-dependently increased in all fasted mice (Fig. 6a,e). To verify the role of PPARα in the process, the hepatic expression of PPARα targeted genes including *MCAD*, cytochrome *P450* and *mHMG-CoAs* was measured by RT-qPCR. Starvation up-regulated the mRNA levels of all the genes measured (Fig. 6b), similar to those caused by RSV treatment (Fig. 3d). In addition, GW6471, a specific PPARα inhibitor, significantly blocked the enhanced activity of the HBV core promoter by fasting in the time course of starvation (Fig. 6d,e), suggesting an essential role of PPARα activation in fasting-induced HBV transcription.

## Discussion

RSV has attracted tremendous attentions in the past years due to its beneficial effects in a number of diseases (e.g. metabolic or cardiovascular diseases, aging, cancer) exerted by its protective functions such as antioxidant properties^15^,^40^, anti-inflammatory^41^ and anti-proliferative functions^42^ respectively. Although RSV was found to have effects on several viral infections, this study is the first time to demonstrate that RSV actually induces HBV replication *in vitro* and *in vivo*. Given the fact that there are increasing successful cases of preclinical trials of RSV treatment on a variety of human diseases^43^,^44^, our data raises a serious concern about the risk of possible side effects of RSV, such as treatment of RSV in patients with metabolic disorders simultaneously infected with HBV. In addition, RSV exists in a number of fruits such as red grapes (50–100 mg/g)^45^ and drinks such as red wine (0.2 mg/l to 5.8 mg/l)^46^ at a notable concentrations. Patients with HBV infection, especially HBV carriers and occult infection patients, would have a risk of HBV recurrence when drinking over-dosed red wine. Given the drinking culture in large populations worldwide, additional studies would be required to determine the guideline amount for red wine drinking in HBV patients.

Our data are consistent with previous finding that RSV is considered as an activator of Sirt1^47^, a member of the mammalian sirtuins or HDAC class III. Ren JH *et al.* have previously demonstrated that overexpression of Sirt1 in HepG2.2.15 cells could increase HBV replication via activating AP-1 factor^48^. However, Curtil C *et al.* observed that activation of Sirt1 by Act3 has little effects on HBV replication without simultaneous stimulation of FXRα factor. The simultaneous activation of FXRα and Sirt1 by respective GW4064 and Act3 is FXRα and PGC-1α-dependent^49^. The discrepancy between the two studies discussed above may be partially due to different cell models and experimental conditions used. In our study, unlike Act3, RSV alone displays a strong ability to promote HBV replication in a Sirt1-PGC-1α and PPARα/RXRα-dependent manner. Sirt1, PGC-1α and PPARα/RXRα proteins appear to constitute a sub-network that modulates the HBV core promoter activity and viral transcription. RSV-induced Sirt1 activation mediates HBV transcription, and inhibition of Sirt1 by NAM attenuates this effect, suggesting that activation of Sirt1 is required for RSV-induced HBV replication. Given the limited effects of Sirt1 overexpression or activation by Act3 on the HBV transcription, the most likely explanation is that RSV has both Sirt1-dependent and independent functions^50^, which can simultaneously activate other signals to augment the function of this sub-network, such as activation of PPARα/RXRα through other pathways.

Fasting induces metabolic responses that allow mammals to survive for a long period of energy deprivation. Dramatic changes in gluconeogenesis and fatty acid oxidation are prominent features of the energy-metabolic responses. Shlomai A *et al.* demonstrated that PGC-1α controls HBV replication through nutritional signals and interestingly, PGC-1α co-activates HNF4α, a key enzyme of gluconeogenesis, to promote HBV replication^37^. However, we think that the role of HNF4α is not conclusive due to lack of loss of function study to demonstrate the necessity of HNF4α in this process. In our study, although the expression of gluconeugenic genes was upregulated after 7-hour fasting, there was no similar change in the gene expression in the time points of prolonged fasting (Fig. 6c), suggesting that the activation of gluconeogenesis is not associated with fasting-induced HBV viral replication. Instead, we found that genes involved in FAO were closely associated with fasting-induced HBV transcription and replication. This was further confirmed by our experiments where inhibition of PPARα, a key enzyme of FAO, alleviated fasting-induced HBV transcription *in vivo*, providing strong evidence that FAO plays an important role in HBV replication in the hosts exposed to nutrient/energy deficiency.

**Figure 7:**
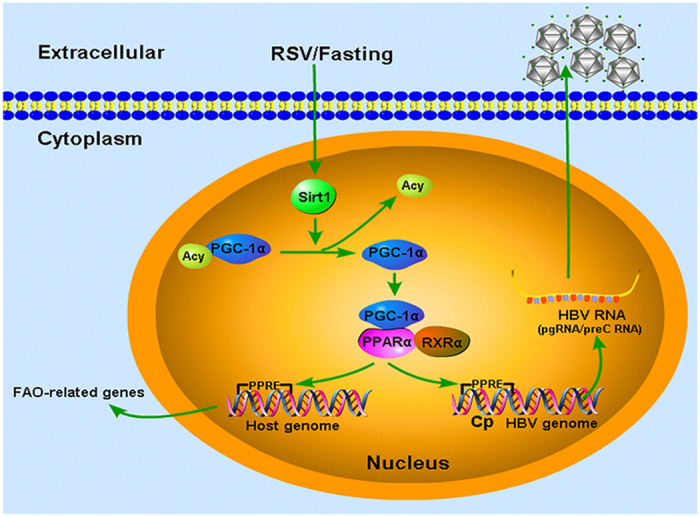
Model for the regulation of RSV on the HBV replication. RSV treatment activates Sirt1, resulting in PGC-1α deacetylation which then coactivates PPARα/RXRα. The activated PPARα/RXRα complex then binds to peroxisome proliferator hormone Response Elements (PPREs) in HBV core promoter (Cp) to enhance HBV transcription, resulting in the increase of HBV DNA replication. In addition, fasting-induced HBV replication also shares the Sirt1- PGC-1α- PPARα/RXRα pathway.

In summary, this study demonstrates that RSV has a strong ability to enhance HBV replication through its core promoter. As shown in Fig. 7, RSV activates Sirt1 and enhances HBV replication in a PGC1α-PPARα/RXRα dependent mechanism, which resembles the pathway of fasting-induced FAO and HBV replication. Supplement of HBV patients with RSV presents a potential risk of hepatitis B recurrence.

## Methods

### Animal experiments

Our animal studies were conducted in strict accordance with the recommendations in the Guide for the Care and Use of Laboratory Animals according to the regulation in the People’s Republic of China. The protocol was approved by the Committee on the Ethics of Animal Experiments of Zhejiang University (Approval No. X1201231). All procedures were made to minimize suffering.

C57BL/6 mice (male, 6–8 weeks old), obtained from Shanghai Laboratory Animal Center (Shanghai, China), were used for animal experiments. For the whole HBV analysis, ten micrograms of HBV plasmid DNA (pHBV) in a volume of PBS equivalent to 10% of the mouse body weight were injected via tail vein on day 0 according to the method^24^. Twenty eight days after the hydrodynamic injection, mice were divided into two groups as follows: RSV group and H2O group. Mice received either RSV (100 mg/kg mouse body weight) or vehicle (water) by oral gavage every day for 2 weeks. The chosen dose was based on a previous study^51^. RSV was re-suspended in water. Mice were sacrificed at 35 and 42 days after injection. Liver tissues and sera were collected for pathologic and biochemical analysis.

For animal imaging experiments, mice were injected with six micrograms of the 1.3× HBV-Cp-Luc construct and analysis was performed 72 h after the hydrodynamic injection^37^. After mice received an intragastric administration of RSV (100 mg/kg/d) or vehicle (water) for 20 minutes, mice were anesthetized with isoflurane, and D-luciferin potassium salt (122796, Perkin Elmer, USA) at 150 mg/kg was then intraperitoneal injection (i.p.) injected. Visualization of luciferase activity was performed on the Lumazone FM1024 equipment (Nippon Roper, Japan). Data analysis including quantification was performed by using the SlideBook™ 4.0 software (Intelligent Imaging Innovations, USA). Luciferase activity of every animal was quantified and expressed as the ratio relative to the baseline activity.

For starvation experiments, male littermates were separated into individual cages at the beginning of each fasting experiment. Fasting was initiated at 5:00 p.m. Mice injected with the 1.3x HBV-Cp-Luc plasmid at 72 h prior to fasting were divided into two groups. The experimental group (n = 6) was fasted (free access to water was allowed) at indicated times (0, 7, 24, 48 h) and allowed to a subsequent 24-hour refeeding. The control group (n = 6) was let for continuous free feeding. The luciferase activity of core promoter was visualized at indicated times. Meanwhile, another 30 mice (divided into 5 groups: 0 h, fasting 7 h, fasting 24 h, fasting 48 h, refed, n = 6) treated as indicated above were killed and liver tissues were collected and immediately frozen in liquid nitrogen for further analysis. To investigate the effect of inhibiting activity of PPARα on the viral core promoter under nutritional deprivation, mice with HBV-Cp-Luc plasmid were administered acute intraperitoneal injection of GW6471 (1 mg/kg, a PPARα antagonist) 30 min prior to defined fasting time points. The dose was chosen based on the previous pilot study^52^. Control animals received the same volume of the vehicle (1% aqueous solution of DMSO). Luciferase activity of every animal was quantified at all time points.

### Plasmids, reagents and antibodies

The 1.3× HBV-Cp-Luc, a “gutless” HBV construct containing a luciferase ORF under the HBV core promoter, was a gift from Yosef Shaul (Department of Molecular Genetics, The Weizmann Institute of Science, Rehovot 76100, Israel)^37^. The plasmid with 1.3mer HBV genomic DNA (GeneBank: U95551.1) (pcDNA3.1 + HBV, pHBV) and the luciferase report vectors (pGL3-Cp, pGL3-S1p, pGL3-S2p containing HBV core promoter (nt 1408–1801), PreS1 promoter(nt 2368–2849) or PreS2 promoter (nt2912–3162) respectively) were constructed in our laboratory according to standard procedures as described^53^. Resveratrol (R5010, Sigma, USA), nicotinamide (72340, Sigma, USA), GW6471 (G5045, Sigma, USA) and Trichostatin A (T1952, Sigma, USA) were purchased from Sigma. The siRNAs for silencing *Sirt1, PGC-1a, c-Jun, FXRα, PPARa, RXRα, HNF4α, C/EBPα* and the scrambled siRNA were purchased from RIBOBIO (Guangzhou, China). The siRNAs sequences are listed in Table 1. The following antibodies were used: anti-Sirt1 (07-131, Merck Millipore, USA), anti-HBcAg (B0586, Dako, Denmark), anti-PGC-1a (H-300) (#sc-13067, Santa Cruz, USA), anti-acetylated-Lysine (#9441, Cell Signaling Technology, USA), anti-GAPDH (2118S, Cell Signaling Technology, USA), Normal Rabbit IgG (12-370, Upstate, USA) and anti-β-actin (4970, Cell Signaling Technology, USA).

### Cell culture, transfection and treatments

Human hepatoma derived cell line HepG2 and HuH7 cells were maintained in Dulbecco’s modified Eagle medium (DMEM) containing 10% FBS. HBV-positive stable cell line HepG2.2.15 cells were cultured in DMEM supplemented with 10% FBS and 400 μg/ml G418. All cells were maintained in a humidified incubator at 37 °C with 5% CO_2_. The transfection was carried out using Lipofectamine 3000 (L3000015, Invitrogen, USA) according to the manufacturer’s instructions. Cell viability was assessed by the MTT assay (Sigma-Aldrich, USA). Resveratrol and nicotinamide (NAM) were used at a concentration of 50 μM and 10 mM, respectively.

### Total RNA extraction and quantitative RT-PCR

Total RNA was extracted using RNAiso Plus (9109, TaKaRa Bio, Japan), and reverse transcription was performed using PrimeScript^™^ RT reagent Kit with gDNA Eraser (Perfect Real Time) (RR047A, TaKaRa Bio, Japan) according to the manufacturer’s instructions. The expression levels of genes of interest were assessed by quantitative PCR (SYBR^®^ *Premix Ex Taq*^™^ II, RR820A, TaKaRa Bio, Japan) using ABI 7900HT Fast Instrument (Applied Biosystems, USA). Samples were processed in triplicate, and analyzed by the 2^−ΔΔCt^ Method. The primers used in the assays were purchased from Sangon Co. Ltd (Shanghai, China) or Applied Biosystems [CYP 4A11 (human), Hs04194779_g1; mHMG-CoAs (human), Hs00985427_m1; ACTB (human), Hs01060665_g1]. Primer sequences are listed in Table 1.

For the detection of pgRNA and preC RNA, the primers used were based on the previous pilot study^54^. In brief, the cDNA product was used in each of three separate amplification reactions with BC1 (5′-GGAAAGAAGTCAGAAGGCAA) as the common 3′ antisense primer and 5′ sense primers: (1) PCP (5′-GGTCTGCGCACCAGCACC) for the specific detection of preC RNA transcripts, (2) PGP (5′-CACCTCTGCCTAATCATC) for monitoring total CP-directed RNA transcription (pgRNA plus preC RNA), and (3) M3 (5′-CTGGGAGGAGTTGGGGGAGGAGATT) for detecting residual HBV DNA contamination. The levels of pgRNA transcripts were calculated by subtracting preC RNA levels from total CP-directed transcription.

### Protein-protein interaction analysis and Western blot

Endogenous protein-protein interaction in cells was examined by co-immunoprecipitation experiments using anti-Sirt1 and anti-PGC-1α antibodies. Cells were lysed with Cell Lysis Buffer (1×) (#9803, Cell Signaling Technology, USA) containing 1 mM PMSF, 10 mM nicotinamide and 10 μM TSA. Lysates were centrifuged (13,000*g*, 4 °C, 10 min) and the supernatants were used for immunoprecipitation. 50 μl of fresh protein G magnetic beads (#LSKMAGG02, Millipore, USA) were added and incubated with 1 μg (2 μL) of anti-Sirt1 or 2.4 μg (12 μl) of anti- PGC-1α for 10 minutes with continuous mixing at room temperature. Cell lysate samples (400 μg) and the immobilized capture antibody were then incubated at 4 °C with continuous mixing overnight. Immunocomplexes were washed several times, denatured with 80 μl 2× Laemmli sample buffer (10 min, 95 °C) and then analyzed by Western blot. The immunoprecipitates were separated by SDS–PAGE and immunoblotted using antibodies against Sirt1 and PGC-1α.

Western blot was performed as follows. Briefly, equal amounts of protein extract were denatured, separated on 10% NuPAGE Bis-Tris Gels and transferred on to PVDF membranes. Afterwards, the membranes were blocked in TBS-T (150 mM NaCl, 10 mM TRIS-HCl pH 7.5, and 0.1% Tween 20) containing 5% (w/v) non-fat dry milk, and incubated with the corresponding primary and secondary antibodies at the indicated dilutions: Sirt1 (1:1000), β-actin (1:3000), GAPDH (1:8000), HBcAg (1:1000), PGC-1a (1:500) and acetylated-Lysine (1:1000). The specific bands were visualized using an ECL detection kit with a ChemiScope 3300 Mini equipment (CLINX, Shanghai, China). The protein levels were quantified by using Image J software (a version of NIH Image, http://rsb.info.nih.gov/ij/).

### PGC-1α acetylation assays

PGC-1α acetylation level was measured by immunoprecipitation of PGC-1α followed by Western blot using anti-acetyl-lysine antibody. PGC-1α and acetylation levels were assayed using specific antibodies for PGC-1α and acetyl-lysine.

### Luciferase ashsay

HepG2 cells in 96-well plates containing 2.0 × 10^4^ cells were transiently transfected with the reporter vector (pGL3-Cp, pGL3-S1p and pGL3-S2p) by using Lipofectamine 3000 according to the manufacturer’s instructions. Transfection mixtures for each well comprised 100 ng of promoter reporter plasmid and 10 ng of plasmid pRL-TK, serving as an internal control to normalize the transfection efficiency. Six hours after transfection, RSV was added to the medium as indicated and cells were incubated for 3 days. Then Firefly and *Renilla* luciferase activities were measured by using a Dual-Glo^®^Luciferase Assay System kit (E2940, Promega, USA) according to the manufacturer’s instructions. The luciferase activity was determined on a GloMax microplate luminometer (Promega, USA).

### NAD+/NADH ratio assay

HepG2.2.1.5 cells were grown to 55% confluency in a 6 cm^2^ tissue culture plate. Cells were subsequently treated with media containing DMSO or RSV (50 μM). After 24 hours of treatment, cells were lifted with trypsin, washed twice with cold PBS and pelleted through centrifugation. NAD + /NADH ratio was performed using the EnzyChrom NAD/NADH Assay Kit (E2ND-48, Bioassay Systems, USA). NAD and NADH contents were normalized by protein concentrations in cell lysates.

### Immunohistochemistry

Liver tissues were fixed in 4% polyformaldehyde and embedded in paraffin. Five μm tissue sections were heated at 55 °C for 2 h, deparaffinized in xylene, rehydrated in a graded series of ethanol, and then incubated in H_2_O_2_ (3%, 10 min). Tissue sections were then blocked with normal goat serum (ZLI-9022, Zhongshan Golden Bridge, Inc., Beijing, China) at 37 °C for 90 min and incubated in primary mouse anti-HBcAg (1:100, ZM-0421, Zhongshan Golden Bridge, Inc., Beijing, China) overnight at 4 °C, followed by incubation in secondary goat anti-mouse antibody (PV-6002, Zhongshan Golden Bridge, Inc., Beijing, China) for 60 min at 37 °C. Sections were then incubated with 3, 3-diaminobenzidine (DAB, ZLI-9018, Zhongshan Golden Bridge Inc., Beijing, China) for 4 min. Samples were then counterstained with hematoxylin for 4 min. Finally, sections were washed again in water, dehydrated, deparaffinized and coverslipped. For negative controls, the primary antibody was replaced by PBS to exclude false positive signals. Images were captured using the Nanozoomer slide scanner (Nanozoomer 2.0-RS; Hamamatsu Photonics, Hertfordshire, UK).

### Detection of serum HBV antigen and DNA

Serum HBsAg and HBeAg levels were detected automatically by Abbott i2000SR using the Architect HBsAg and HBeAg Reagent kits (Abbott Diagnostics, Abbott Park, IL, USA). Serum HBV DNA copies were measured using the Fluorescence Quantitative PCR Detection Kit for Hepatitis B Virus DNA (ACON Biotech Co. Ltd, Hangzhou, China). The HBsAg, HBeAg and HBV DNA in the culture medium were measured similarly. All of these assays were conducted following the manufacturers’ instructions.

### Statistics

Data were analyzed using GraphPad Prism v5.0a (GraphPad Software, Inc., SanDiego, USA). Data were presented as mean ± SEM. Statistical significance of the differences was determined using Student *t* test. Differences were considered significant when *P* < 0.05.

## Additional Information

**How to cite this article**: Shi, Y. *et al.* Resveratrol enhances HBV replication through activating Sirt1-PGC-1a-PPARa pathway. *Sci. Rep.* **6**, 24744; doi: 10.1038/srep24744 (2016).

## Supplementary Material

Supplementary Information

## Figures and Tables

**Table 1:** Primer and siRNAs sequences used in the study.

primer name	gene ID	forward primer (F)(5′ → 3′)	reverse primer(R)(5′ → 3′)
HNF4α (human)	NM_001287182	CGAAGGTCAAGCTATGAGGACA	ATCTGCGATGCTGGCAATCT
FXRα (human)	NM_001206993	TCGCAATACAGCAATGTTCAG	GCTACCTCAGTTTCTCCCTGGT
c-Jun (human)	NM_002228	GCAGCCCAAACTAACCTCAC	GTAGCCATAAGGTCCGCTCTC
C/EBPα (human)	NM_004364	AGGTTTCCTGCCTCCTTCC	CCCAAGTCCCTATGTTTCCA
PGC-1α (human)	NM_013261	TGCTGAAGAGGCAAGAGACA	CACACACGCACACTCCATC
PPARα (human)	NM_001001928	TTCGCAATCCATCGGCGAG	CCACAGGATAAGTCACCGAGG
RXRα (human)	NM_002957	CGAGAATGAGGTGGAGTCG	AATGTTGGTGACAGGGTCGT
actin (human)	NM_001101	GTGGCCGAGGACTTTGATTG	AGTGGGGTGGCTTTTAGGATG
Sirt1 (human)	NM_012238	GCTGGCCTAATAGAGTGGCAA	CTCAGCGCCATGGAAAATG
MCAD (human)	NM_000016	GGAAGCAGATACCCCAGGAAT	AGCTCCGTCACCAATTAAAACAT
actin (mouse)	NM_007393	GTGACGTTGACATCCGTAAAGA	GCCGGACTCATCGTACTCC
Sirt1 (mouse)	NM_001159589	ATGACGCTGTGGCAGATTGTT	CCGCAAGGCGAGCATAGAT
MCAD (mouse)	NM_007382	AGGGTTTAGTTTTGAGTTGACGG	CCCCGCTTTTGTCATATTCCG
CYP 4A14 (mouse)	NM_007822	GGAGCAATATACGAGTCCTGC	CAGAGTCCGCCATGATTTTGA
mHMG-CoAs (mouse)	NM_008256	GAAGAGAGCGATGCAGGAAAC	GTCCACATATTGGGCTGGAAA
FP2ase (mouse)	NM_019395	AGTCGTCCTACGCTACCTGTG	GGGGATCGAAACAGACAACAT
G6Pase (mouse)	NM_008061	CGACTCGCTATCTCCAAGTGA	GTTGAACCAGTCTCCGACCA
PEPCK-c (mouse)	NM_011044	AGCATTCAACGCCAGGTTC	CGAGTCTGTCAGTTCAATACCAA
siRNA name	gene ID	sense(5′ → 3′)	antisense(5′ → 3′)
Sirt1	23411	CCAUCUCUCUGUCACAAAUTT	AUUUGUGACAGAGAGAUGGTT
c-Jun	3725	GAACAGGUGGCACAGCUUA	CUUGUCCACCGUGUCGAAU
FXRα	9971	GGACCAUGAAGACCAGAU	CCUGGUACUUCUGGUCUAA
RXRα	6256	GCGCCAUCGUCCUCUUUAA	CGCGGUAGCAGGAGAAAUU
HNF4α	3172	CCACAUGUACUCCUGCAGAU	GGUGUACAUGAGGACGUCUA
C/EBPα	1050	GGAGCUGACCAGUGACAAU	CCUCGACUGGUCACUGUUA
PGC-1α	10891	GAGAAUUCAUGGAGCAAUA	CUCUUAAGUACCUCGUUAU
PPARa	5465	GGAGCAUUGAACAUCGAAU	CCUCGUAACUUGUAGCUUA
negative control		UUCUCCGAACGUGUCACGUTT	ACGUGACACGUUCGGAGAATT
