# Supplementary material for: Resveratrol enhances HBV replication through activating Sirt1-PGC-1α-PPARα pathway
Source: Sci Rep. 2016 Apr 21;6:24744. doi: 10.1038/srep24744 (PMC4838842; doi:10.1038/srep24744)
Supplement: Supplementary Information [file srep24744-s1.doc]

**Resveratrol enhances HBV replication through activating Sirt1-PGC-1α-PPARα pathway**

Yixian Shi^1^, Yongjun Li^1^, Chenjie Huang^1^, Lixiong Ying^2^, Jihua Xue^3^, Haicong Wu^4^, Zhi Chen^1^, and Zhenggang Yang^1,*^

^1^State Key Lab of Diagnostic and Treatment of Infectious Diseases, Collaborative Innovation Center for Diagnosis and Treatment of Infectious Disease, the First Affiliated Hospital, Zhejiang University School of Medicine, Hangzhou 310003, China.

^2^Department of Pathology, the First Affiliated Hospital, College of Medicine, Zhejiang University, Hangzhou 310003, China.

^3^Department of Infectious Disease, the First Affiliated Hospital of Anhui Medical University, Hefei 230022, Anhui, China.

^4^Department of Hepatobiliary Medicine, Fuzhou General Hospital of Nanjing Military Command, Fuzhou 350025, Fujian, China.

*Correspondence and requests for materials should be addressed to Z.Y. (email: [yangzg@zju.edu.cn](mailto:yangzg@zju.edu.cn)).


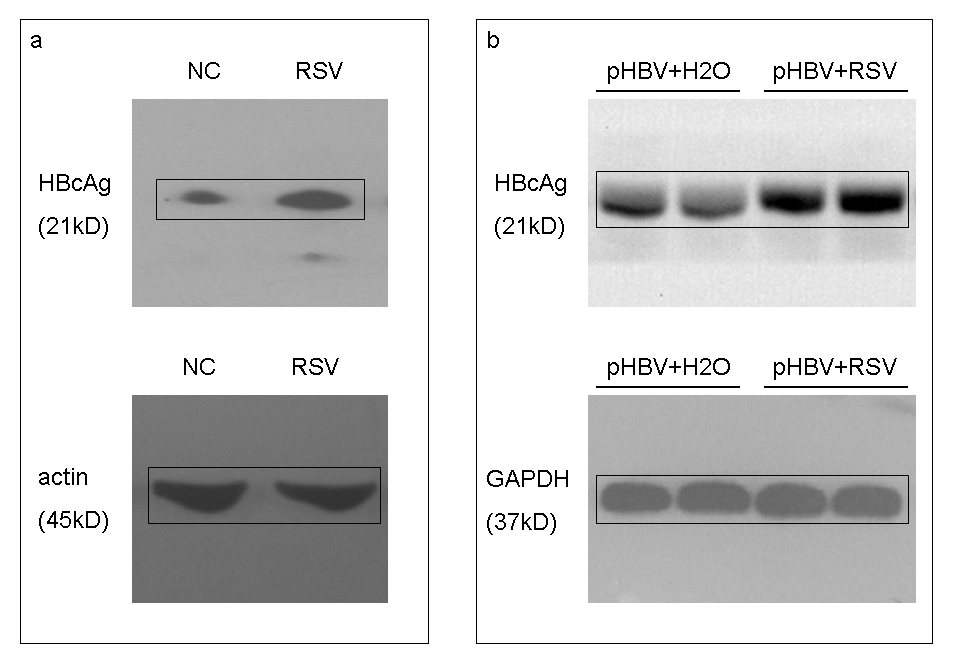


**Supplementary** **Figure 1: The full-length blots of Figure 2.** (a) The full-length blots of Figure 2a. (b) The full-length blots of Figure 2b.


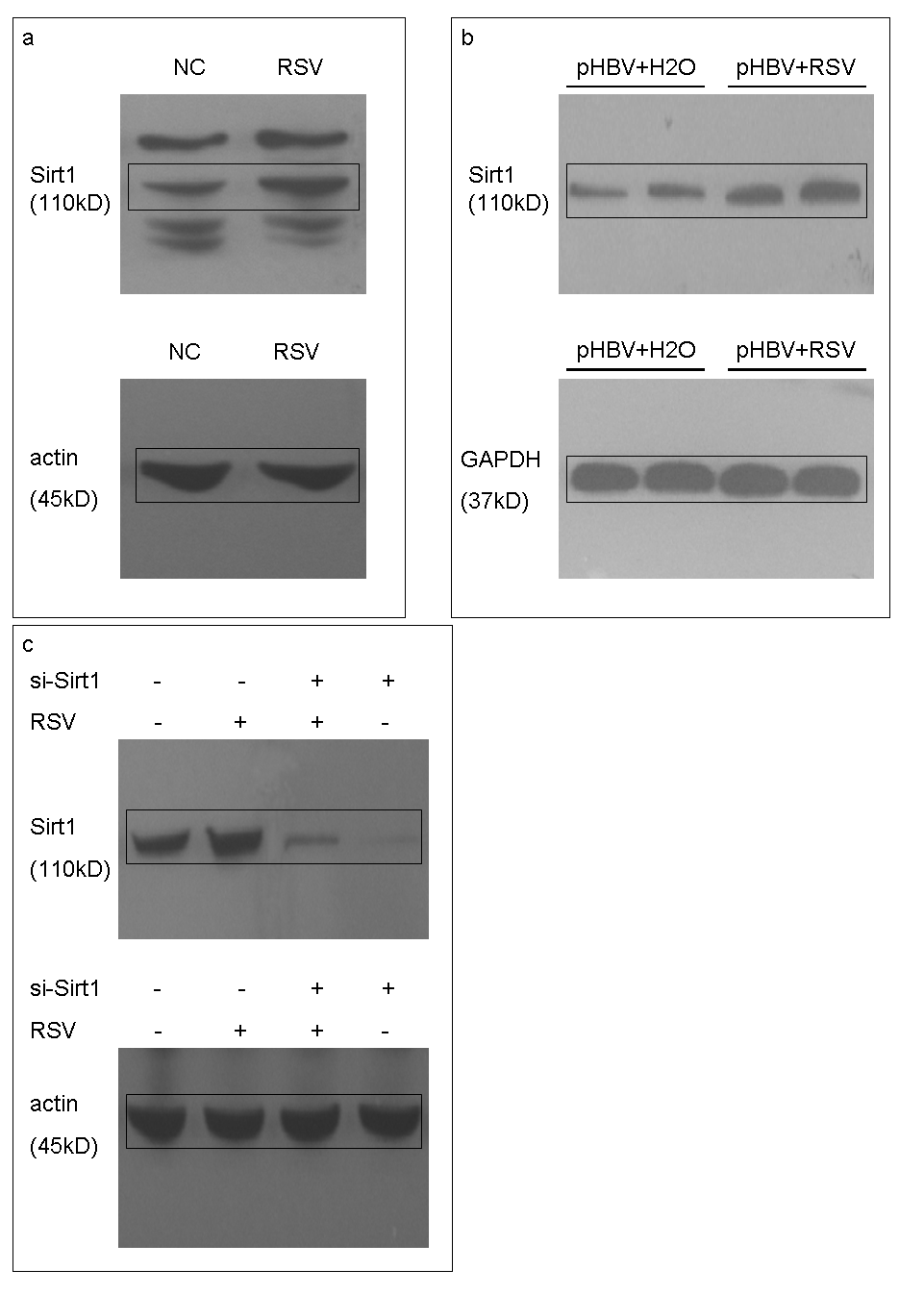


**Supplementary** **Figure 2: The full-length blots of Figure 4.** (a) The full-length blots of Figure 4a. (b) The full-length blots of Figure 4b. (c) The full-length blots of Figure 4d.


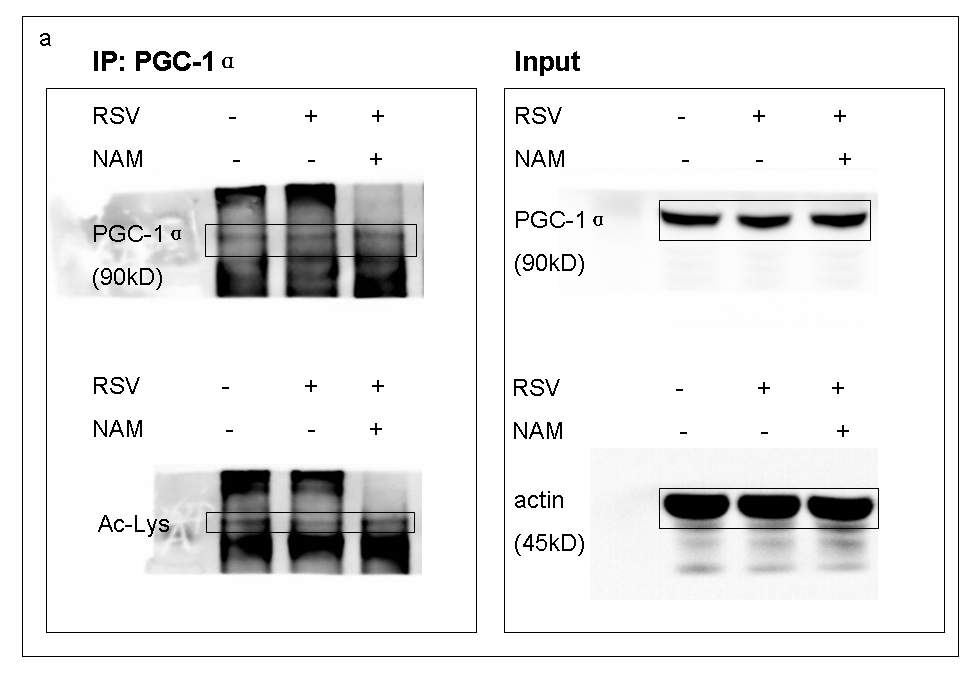


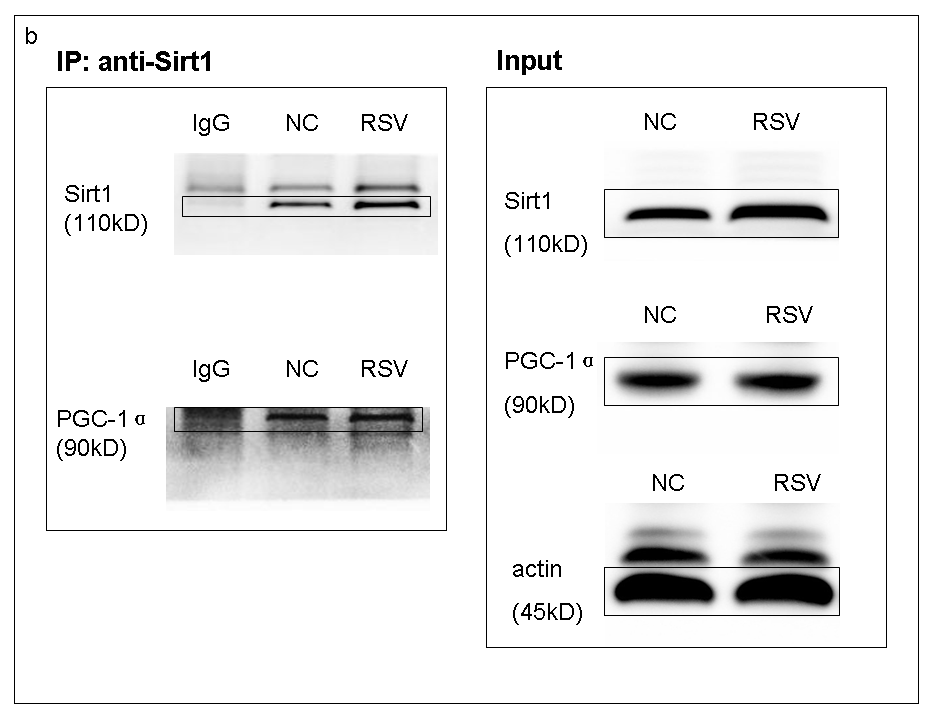


**Supplementary** **Figure 3: The full-length blots of Figure 5.** (a) The full-length blots of Figure 5a. (b) The full-length blots of Figure 5b.
